# Ultrasound evaluation of diaphragmatic mobility in patients with idiopathic lung fibrosis: a pilot study

**DOI:** 10.1186/s40248-018-0159-y

**Published:** 2018-12-14

**Authors:** Andrea Boccatonda, Valentina Decorato, Giulio Cocco, Stefano Marinari, Cosima Schiavone

**Affiliations:** 1 Unit of Internistic Ultrasound, Department of MedicineScience of Aging G. D’Annunzio University, Cesi –Met, via Luigi Polacchi, 66100 Chieti, (CH) Italy; 2 Unit of Pneumology, SS Annunziata University Hospital, Chieti, Italy

**Keywords:** Lung, Ultrasound, Interstitial lung disease, Diaphragm

## Abstract

**Background:**

Some previous works have tested LUS use in diagnosing and monitoring interstitial lung diseases. B-lines are main sonographic signs for interstitial diseases. Increasing evidences suggest that dyspnea and impaired exercise capacity in IPF patients can be related, at least in part, to respiratory muscle dysfunction, in particular to diaphragm functionality. Both B-mode and M-mode ultrasound techniques have been employed to assess diaphragm excursion (DE), which measures the distance that the diaphragm is able to move during the respiratory cycle.

**Methods:**

The main objective of this case-control study was to evaluate if differences exist between diaphragmatic excursions in IPF patients and in a control group of healthy subjects. Secondary objectives were to evaluate possible correlations among respiratory excursions, anthropometric parameters and respiratory function parameters. All patients performed spirometry and body plethysmography (PC). Diaphragm was examined by ultrasound imaging in B-MODE, and respiratory excursions were evaluated in M-MODE. Examination consisted of 3 measurements of the inspiratory phase at rest and after deep inspiration.

**Results:**

Twelve patients with IPF and 12 healthy subjects were enrolled. There were no significant differences between respiratory excursions in patients and controls during spontaneous breathing, while there was a statistically significant difference between the mean values of the deep respiratory excursion in the two groups (*p* value < 0.001). There was a positive correlation between respiratory excursion with normal breath and chest circumference in controls (*p* = 0.034; *R* = 0.614) and in patients (*p* = 0.032; *R* = 0.37), but this relationship was not found even in subjects in deep breathing. A positive correlation was found between FVC values and diaphragmatic motility both at rest and in deep breathing in fibrotic patients.

**Conclusions:**

Diaphragmatic mobility is lower in IPF patients than in healthy controls, especially during deep inspiration. The correlation between reduced FVC and diaphragmatic excursion values in IPF patients can be of interest, since it could represent an index of functional respiratory function performed by a non-invasive, low-cost, simple and reliable imaging technique, such as LUS.

## Background

Idiopathic pulmonary fibrosis (IPF) is a progressive idiopathic fibrotic lung disease, characterized by a poor prognosis. The main symptom in IPF patients is increasing dyspnea on exertion. Diagnosis is based on clinical history and examination, serological assays, lung functioning tests, and computed tomography (CT). Furthermore, invasive procedures such as bronchoscopy or transbronchial biopsy, and thoracic surgery are often necessary to differential diagnosis. The Fleischner Society recently published consensus about radiographic criteria for IPF, such as reticular opacities with honeycombing, usually associated with traction bronchiectasis, and ground glass opacity usually admixed with reticular abnormality and honeycombing [1]. Such abnormalities are characteristically basal and peripheral, although they are often patchy [1].

In last years, lung ultrasound (LUS) has been employed as a new diagnostic tool to study lung and chest diseases, in particular in the emergency setting. Some previous works have tested LUS use in diagnosing and monitoring interstitial lung diseases [2–7]. B-lines are main sonographic signs for interstitial diseases [5, 7–10]. In interstitial lung diseases, B lines are characterized by a nonhomogeneous distribution, together with some alterations in the pleural line (fragmentation, irregularity and swelling) [2, 7, 10].

Increasing evidences suggest that dyspnea and impaired exercise capacity in IPF patients can be related, at least in part, to respiratory muscle dysfunction [11, 12], in particular to diaphragm functionality.

Recently, diaphragmatic ultrasound evaluation has gained importance as a safe, radiation free, bed-side tool to study diaphragm function and predicting weaning outcome [13]. Ultrasound can provide both morphological and functional information, then allowing to repeat measurements over time. Both B-mode and M-mode ultrasound techniques have been employed to assess diaphragm excursion (DE), which measures the distance that the diaphragm is able to move during the respiratory cycle [14, 15]. This sonographic parameter seems to be the optimal predictor of lung volume reduction. The main objective of our study was to evaluate if differences exist between DE in IPF patients and in a control group of healthy subjects. Secondary objectives were to evaluate possible correlations between respiratory excursions, anthropometric parameters and respiratory function parameters.

## Material and Methods

### Study population

Patients diagnosed with IPF related to the pulmonary fibrosis center of the SS Annunziata Clinical Hospital of Chieti were enrolled. IPF diagnosis was based on clinical findings, respiratory function tests, high-resolution chest CT scan, bronchioloalveolar lavage and, in some cases, pulmonary biopsy. Patients were excluded if they had ongoing lung infections and if they had neuromuscular disorders associated with pulmonary fibrosis. In addition, healthy volunteers were enrolled. Each patient provided written informed consent to participate. This study was performed under the Good Clinical Practice regulations and the Declaration of Helsinki (Hong Kong 1989).

### General examination and functional measurements

Each patient was subjected to clinical examination, anthropometric measurements including weight, height, abdomen and thorax circumference. Furthermore, all patients performed spirometry and body plethysmography (PC) by using a plectrism-spirometer MasterScope Body JAEGER (CareFusion, Hoechberg German) to obtain the following variables: forced vital capacity (FVC), total lung capacity (TLC), forced expiratory volume in the 1^st^ second/ forced vital capacity (FEV_1_ / FVC max), vital capacity (VC) and diffusion Lung CO (DL_CO_).

### Ultrasound technique

Diaphragmatic motility was evaluated by a portable Aloka Prosound ultrasound, by using a 2–5 MHz convex probe. Patients were examined in supine position, with a minimum saturation of O_2_ > 94%. A right ascending subcostal scan in the area between the anterior axillary line and the midclavicolar line was employed. Diaphragm was examined in B-MODE, and respiratory excursions were evaluated in M-MODE. Examination consisted of 3 measurements of the inspiratory phase at rest and after deep inspiration [16]. Measurements in patients and healthy controls were performed by the same operator, freezing the image of the diaphragmatic curve during the respiratory cycle and measuring the distance from the base of the curve to the apex. The operator was not aware of the state of health of the subject examined during LUS.

### Statistical analysis

The statistical analysis of the data was performed using SPSS software (IBM analytics, Armonk USA), the graphs were created using Graphpad Prism 5 (Graphpad Software Inc., La Jolla California USA). *P* < 0.05 were considered to be statistically significant.

## Results

Twelve patients with idiopathic pulmonary fibrosis and 12 healthy subjects were enrolled for the study. Baseline characteristics are shown in Table 1.

**Table 1:** Anthropometric and lung function measurements

	Controls	Patients	*P*
Age	66.5 ± 11	71.6 ± 5.5	0.216
BMI	24.0 ± 4.0	28.1 ± 2.4	0.006
Smoking (%)	5 (41.7)	10 (83.3)	0.035
CT (cm)	81.3 ± 10.3	97.8 ± 4.6	< 0.001
CA (cm)	82.3 ± 12.8	96.9 ± 8.5	0.003
FVC	111.6 ± 35.2	72.5 ± 7.5	0.001
TLC	112.9 ± 27.8	55.4 ± 7.5	< 0.001
VC	117.8 ± 13.4	72.0 ± 9.0	< 0.001
DL_CO_ mL/min/mm Hg	82.1 ± 24.8	37.8 ± 8.3	< 0.001
FEV_1_/FVC max	85.4 ± 7.1	85.1 ± 5.7	0.904
DE (normal breath) (cm)	1.5 ± 0.6	1.7 ± 0.7	0.503
DE (deep inspiration) (cm)	6.3 ± 1.3	3.7 ± 1.1	< 0.001

*Abb*. *BMI*: body mass index; *CT*: chest circumference; *CA*: circumference of abdomen; *FVC*: forced vital capacity; *TLC*: total lung capacity; *FEV1/FVC max*: forced expiratory volume in the 1^st^ second/ forced vital capacity; *VC*: vital capacity; *DLCO*: diffusion Lung CO; *DE*: diaphragmatic excursion

There were no significant differences between respiratory excursions in patients and controls during spontaneous breathing, while there was a statistically significant difference between the mean values of the deep respiratory excursion in the two groups (*p* < 0.001).

**Fig. 1:**
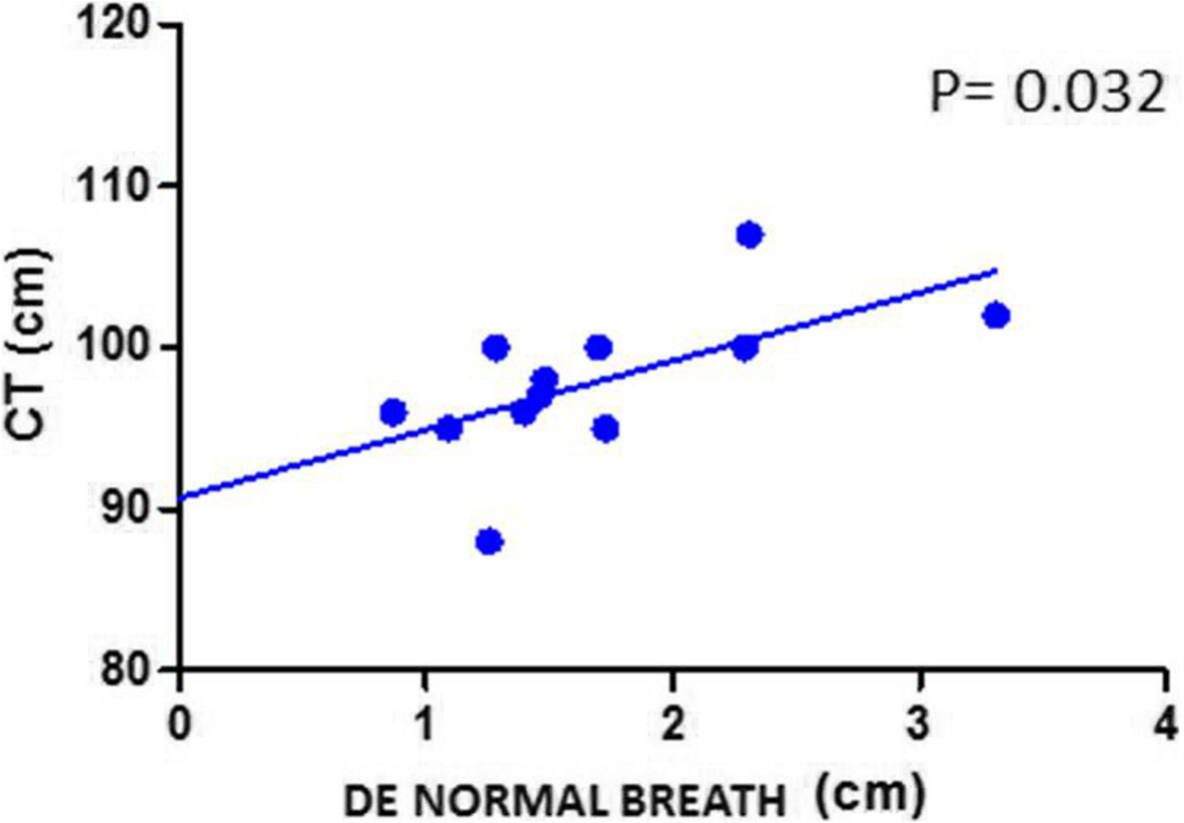
Correlation between CT and DE during normal breathing in IPF patients. *CT*: chest circumference; *DE*: diaphragmatic excursion

**Fig. 2:**
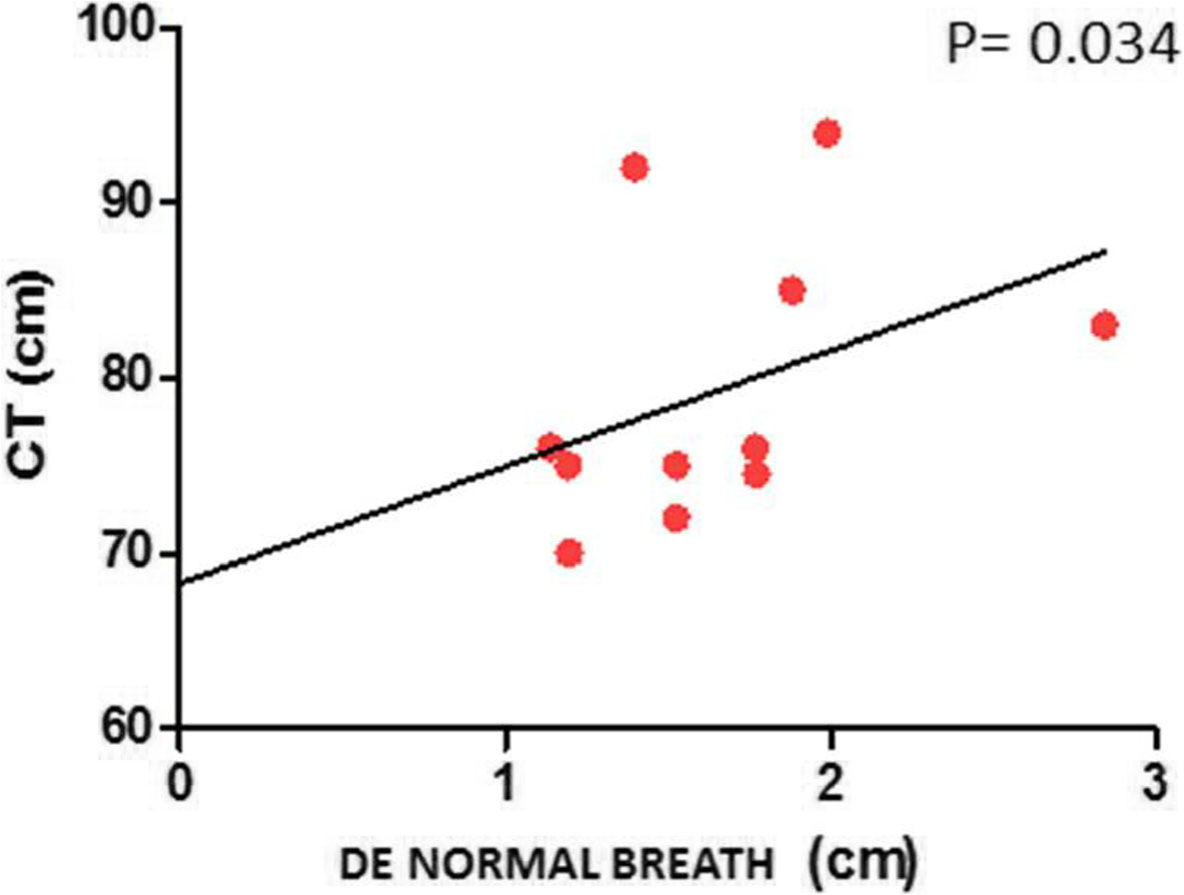
Correlation between CT and DE during normal breathing in healthy subjects. *CT*: chest circumference; *DE*: diaphragmatic excursion

Pearson’s correlations between respiratory excursion and physical characteristics showed a positive correlation between respiratory excursion with normal breath and chest circumference in controls (p 0.034; R 0.614) and in patients (p 0.032; R 0.37) (Figs 1 and 2), but this relationship was not found even in subjects with deep breathing (Table 2).

**Table 2:** Correlations between diaphragmatic excursion values and anthropometric and lung function measurements

		Patients		Controls	
		Normal Breath	Deep inspiration	Normal Breath	Deep inspiration
BMI	R	− 0.41	0.06	0.59	0.25
	*p*	0.17	0.83	0.04	0.43
CT	R	0.37	−0.01	0.61	0.22
	*p*	0.03	0.95	0.03	0.49
CA	R	−0.52	− 0.12	0.55	0.03
	*p*	0.07	0.69	0.06	0.91
FVC	R	0.53	0.56	0.09	0.22
	*p*	0.05	0.05	0.78	0.49
TLC	R	−0.04	−0.13	−0.59	0.31
	*p*	0.88	0.68	0.40	0.32
VC	R	0.19	−0.11	0.07	0.17
	*p*	0.55	0.73	0.82	0.59
DLCO	R	−0.33	−0.18	0.05	−0.22
	*p*	0.29	0.57	0.85	0.46
FEV1% VC MAX	R	−0.02	−0.39	−0.07	0.69
	*p*	0.94	0.19	0.82	0.01

*Abb*. *BMI*: body mass index; *CT*: chest circumference; *CA*: circumference of abdomen; *FVC*: forced vital capacity; *TLC*: total lung capacity; *FEV1/FVC max*: forced expiratory volume in the 1^st^ second/ forced vital capacity; *VC*: vital capacity; *DLCO*: diffusion Lung CO; *DE*: diaphragmatic excursion

**Fig. 3:**
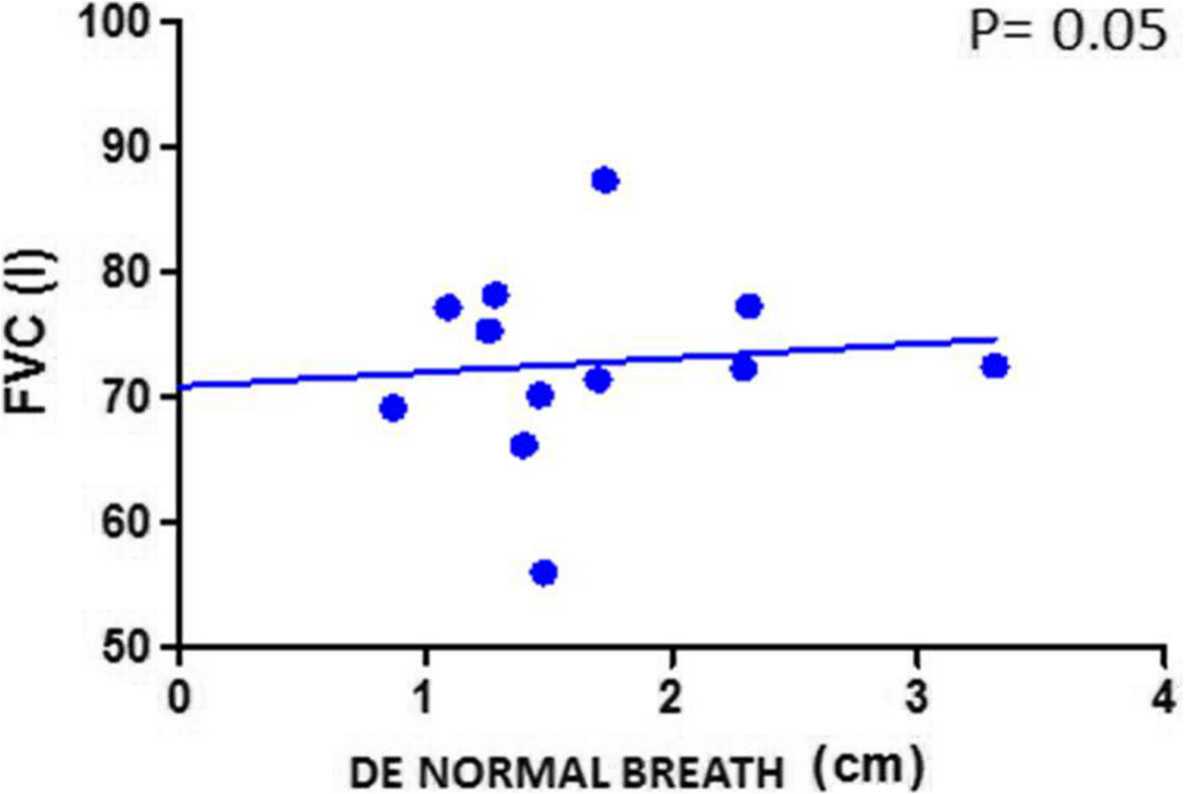
Correlation between FVC and DE during normal breathing in IPF patients. *FVC*: forced vital capacity; *DE*: diaphragmatic excursion

**Fig. 4:**
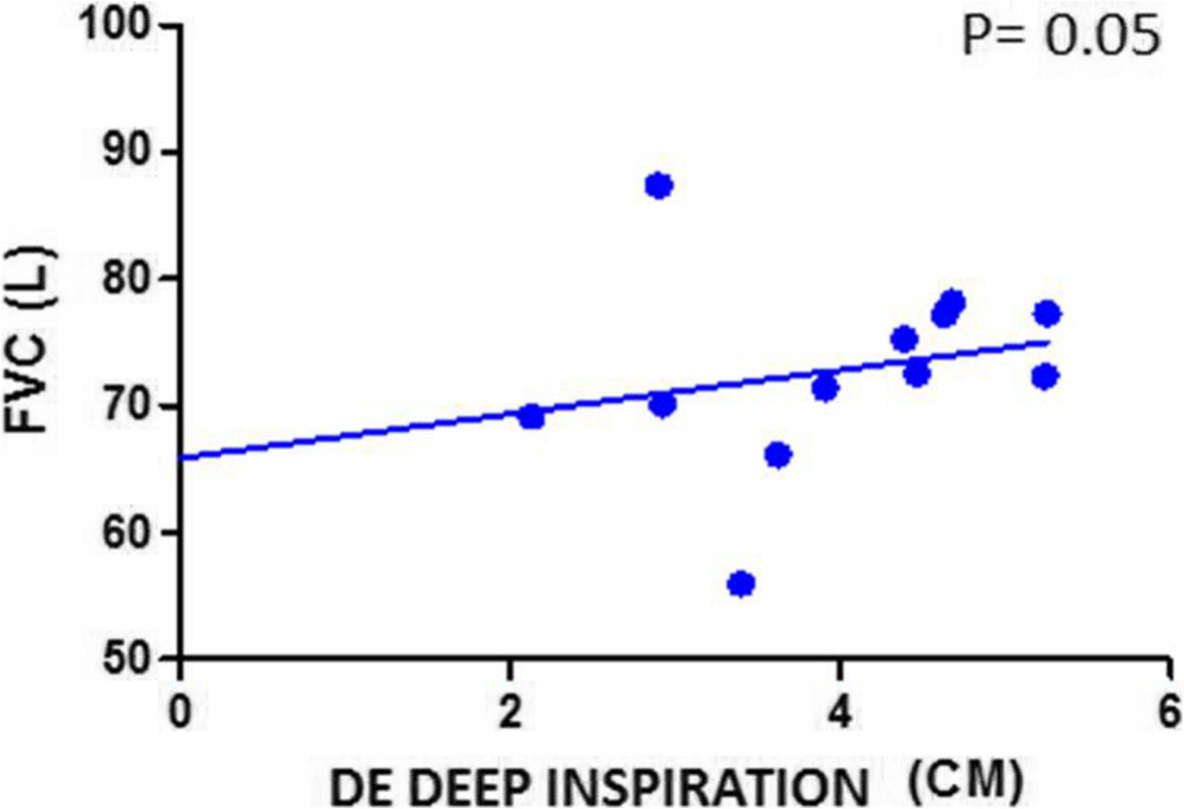
Correlation between FVC and DE during deep inspiration in IPF patients. *FVC*: forced vital capacity; *DE*: diaphragmatic excursion

Finally, a positive correlation was found between FVC values and diaphragmatic motility both at rest and with deep breathing in fibrotic patients (Figs. 3 and 4).

## Discussion

IPF patients are characterized by a restrictive ventilatory defect [17]. Reduction of lung compliance has been demonstrated to be the key to restriction, because both chest wall compliance and respiratory muscle strength are mostly preserved [18]. The diaphragm is a dome-shaped muscle between the thoracic and abdominal cavities [19], and it is responsible for approximately 75% of the respiratory movements. Diaphragmatic weakness is related to decreased or delayed excursion on deep breathing [23]. Our results demonstrate that IPF patients display a significant reduction in mean diaphragmatic excursion compared to control subjects. This reduction is determined by reduced lung volumes due to fibrotic lung, compared to the healthy one. This alteration is detectable in deep breathing maneuver only, which is strongly limited by fibrotic alteration. These data are in agreement with the results of the work of Santana et al. demonstrating that diaphragmatic mobility is lower in interstitial lung disease patients than in healthy controls [21]. Otherwise, a previous work of He et al. found no differences in diaphragmatic motion measured on M-mode sonographic images between IPF patients and healthy controls [22]. Moreover, previous studies investigating DE through LUS in some categories of respiratory patients, such as COPD, failed to demonstrate any relationship with the parameters of respiratory function, but found a close relation with BMI [20]. Otherwise, thickness of the diaphragm in COPD patients was related lung hyperinflation indices and dynamic volumes [20]. These differences can be explained by different study setting or by a real pathological mechanisms due to those diseases.

Furthermore, the lack of difference in diaphragmatic excursion during normal breath can be explained by the fact that fibrotic lung, although less distensible, can still ensure a current volume at rest and, therefore, an excursion within limits.

Furthermore, reduced diaphragmatic excursion is correlated to FVC, a fibrotic evolution index, in IPF patients both in normal and in deep inspiration; this data demonstrates that not only ultrasound can demonstrate a proportional reduction of lung volumes by evaluating diaphragmatic excursion in deep exhalation, but even that breath inhalation is adapted to this reduction, because lung volumes are proportionally reduced in comparison with CVF impairment.

Moreover, our results demonstrate a direct correlation between diaphragmatic excursion and chest circumference, thus confirming the inter-individual variability correlated to the anthropometric parameters characterizing this type of measurement.

Our study is characterized by some limitations. First of all, the small sample size can raise some concerns. Otherwise, our results may be very significant given the smallness of the samples analyzed and could be considered to design a future case-control study with a greater number of patients. Spirometry and body PC were performed with the patients in an orthostatic position, while ultrasound measurements were obtained holding patients in a supine position. In this setting, the role played by the abdomen and the abdominal muscles can differ, leading to a reduction of the total inspired volume due to increased air trapping and to a change in the end expiratory volume. Correlations of diaphragm measurements and lung volumes could be affected by this bias. However, we decided to employ this sonographic technique to analyze also patients who cannot tolerate orthostatic position. Patient and control groups were correlated by age, while they showed a significant difference in BMI values and smoking habits, potentially confounding study results. Indeed, BMI and obesity have been shown to be modulator of diaphragm muscle shape and size of the ring of its insertion [24].

## Conclusion

Diaphragmatic mobility is lower in IPF patients than in healthy controls, especially during deep inspiration. The correlation between reduced FVC and diaphragmatic excursion values in IPF patients can be of interest, since it could represent an index of functional respiratory function performed by a non-invasive, low-cost, simple and reliable imaging technique, such as LUS.

## Abbreviations

BMI: Body mass index
CA: Circumference of abdomen
CT: Chest circumference
CT: Computed tomography
DE: Diaphragm excursion
DLCO: Diffusion Lung CO
FEV1/FVC max: Forced expiratory volume in the 1^st^second/ forced vital capacity
FVC: Forced vital capacity
IPF: Idiopathic pulmonary fibrosis
LUS: Lung ultrasound
PC: Plethysmography
TLC: Total lung capacity
VC: Vital capacity
